# Adrecizumab, a non-neutralizing anti-adrenomedullin antibody, improves haemodynamics and attenuates myocardial oxidative stress in septic rats

**DOI:** 10.1186/s40635-019-0255-0

**Published:** 2019-05-15

**Authors:** Alice Blet, Benjamin Deniau, Christopher Geven, Malha Sadoune, Anaïs Caillard, Paul-Robert Kounde, Evelyne Polidano, Peter Pickkers, Jane-Lise Samuel, Alexandre Mebazaa

**Affiliations:** 1 Department of Anesthesia, Burn and Critical Care, University Hospitals Saint-Louis – Lariboisière, AP-HP, Paris, France; 2 UMR-S 942, Inserm, Paris, France; 3 Paris Diderot University, Sorbonne Paris Cité, Paris, France; 4 Department of Intensive Care Medicine, Radboud Center for Infectious Diseases (RCI), Radboud University Medical center, HP: 710, PO Box 9101, 6500 HB Nijmegen, The Netherlands

## Abstract

**Background:**

Sepsis still represents a major health issue, with persistent high morbidity and mortality rates. Cardiovascular dysfunction occurs frequently during sepsis. Adrenomedullin has been identified as a key mediator in vascular tone regulation. A non-neutralizing anti-adrenomedullin antibody, Adrecizumab, may improve haemodynamic dysfunction during caecal ligation and puncture-induced septic shock in a murine model. Our objective was to determine the role of Adrecizumab on haemodynamics in a rat model of sepsis.

**Methods:**

For the induction of sepsis, caecal ligation and puncture were performed in Wistar male rats. Single blinded administration of Adrecizumab (2 mg/kg) or placebo was injected i.v. 24 h after the surgery, and norepinephrine was infused as the standard of care. There were > 7 animals per group. Invasive blood pressure and cardiac function (by echocardiography) were assessed until 3 h after Adrecizumab injection.

**Results:**

A single therapeutic injection of Adrecizumab in septic rats induced rapid haemodynamic benefits with an increase in systolic blood pressure in septic-Adrecizumab rats versus untreated-septic rats (*p* = 0.049). The shortening fraction did not differ between the untreated-septic and septic-Adrecizumab groups. However, cardiac output increased during the 3 h after a single dose of Adrecizumab compared to untreated septic rats (*p* = 0.006). A single dose of Adrecizumab resulted in similar haemodynamics to the continuous administration of norepinephrine.

Three hours after a single injection of Adrecizumab, there was no change in the inflammatory phenotype (TNFα, IL-10) in the hearts of the septic rats. By contrast, 3 h after a single Adrecizumab injection, free-radical production decreased in the hearts of septic-Adrecizumab vs untreated septic rats (*p* < 0.05).

**Conclusions:**

In a rat model of sepsis, a single therapeutic injection of Adrecizumab rapidly restored haemodynamic parameters and blunted myocardial oxidative stress. Currently, a proof-of-concept and dose-finding phase II trial (Adrenoss-2) is ongoing in patients with septic shock and elevated concentrations of circulating bio-adrenomedullin.

**Electronic supplementary material:**

The online version of this article (10.1186/s40635-019-0255-0) contains supplementary material, which is available to authorized users.

## Background

Despite advances in resuscitation and infectious disease management, sepsis remains one of the leading causes of death worldwide [1, 2]. Sepsis is characterized by disturbed vascular integrity and the presence of life-threatening organ dysfunction due to a dysregulated response of the body to infection [2]. Today, vasopressor therapy is one of the cornerstones of sepsis treatment; however, vasopressor use does not restore vascular integrity and could even lead to harmful effects and impair prognosis [3].

Adrenomedullin (ADM), a 52 amino acid peptide hormone [4, 5], has been proposed as a pivotal mediator of vascular dysfunction in sepsis [6, 7]. On the one hand, ADM can act as a vasodilator, decrease peripheral vascular resistance, and increase cardiac output [8]. On the other hand, ADM has beneficial effects, as it reduces capillary hyperpermeability in preclinical studies with models of septic shock [9, 10]. Recently, a model has been proposed that explains these different activities of ADM as a function of its compartmental localization [11]: in the interstitium, ADM acts on vascular smooth muscle cells to induce vascular relaxation, whereas in the blood circulation, ADM promotes the stabilization of the endothelial barrier. The ADM pathway acts through heterodimeric receptor complexes called “ADM receptors”, which are composed of a calcitonin-receptor-like receptor (CRLR) and receptor activity-modifying proteins (RAMP2 or RAMP3) [12].

In patients with sepsis and septic shock, an elevated plasma concentration of biologically active ADM (bio-ADM) is associated with disease severity and organ dysfunction, and it is a strong prognosticator for 28-day mortality [13–16]. Of interest and related to its vascular effects, high plasma concentrations of bio-ADM are correlated with vasopressor use [13–16].

Therefore, modulation of ADM activity could have therapeutic potential during sepsis to restore haemodynamics and improve clinical outcome [17]. Adrecizumab (HAM 8101) is a humanized non-neutralizing monoclonal antibody directed against the N-terminus of ADM that only partially inhibits ADM activity. Adrecizumab i.v. administration leads to an immediate and substantial increase in plasma ADM concentration, thereby enhancing the endothelium-stabilizing effect of ADM. Adrecizumab acts by decreasing ADM concentration in the interstitium and neutralizing the excess ADM in plasma [18]. In a mouse model of sepsis (caecal ligation and puncture, CLP), preventive treatment by Adrecizumab increased survival, while other antibodies directed against different epitopes of ADM (causing greater or complete inhibition of ADM signalling) did not [18, 19]. In addition, Adrecizumab preventive treatment led to numerous improvements, including reduced catecholamine and fluid requirements and improved renal function [20]. The therapeutic administration of Adrecizumab has not been tested in a preclinical model of sepsis. This study is the first delayed or therapeutic application of ADZ in a preclinical model.

Given the role of ADM in vasodilation and capillary leakage, the objective of this work was to explore the haemodynamic, inflammatory, and myocardial oxidative stress responses to therapeutic treatment with Adrecizumab, which induced partial inhibition of ADM, in a sepsis model in rats.

We hypothesized that by this treatment, a rapid and sustained beneficial response could be achieved in septic shock.

## Materials and methods

### Animals and sepsis model

Two-month-old male Wistar rats weighing 350 to 450 g were obtained from Janvier (St. Berthevin, France). All experiments were conducted in accordance with the National and European Institutes of Health guidelines for the use of laboratory animals and were approved by the local animal research ethics committee (Lariboisière-Villemin, Paris, France) (77-2014-ceea9).

All animals were anesthetized using ketamine hydrochloride (90 mg/kg) and xylazine (9 mg/kg) intraperitoneally. For the induction of polymicrobial sepsis, CLP was performed as previously described [21]. A ventral midline incision (1 cm) was made to allow exteriorization of the caecum. The caecum was then ligated just below the ileocecal valve and punctured once with an 18-gauge needle. The abdominal cavity was closed in two layers, and rats were given fluid resuscitation (3 mL/100 g of body weight of saline injected subcutaneously). A sham operation was performed by isolating the caecum without ligation or puncture. An injection of 75 μg/kg of buprenorphine intraperitoneally was administered for analgesic purposes in the preoperative period. Pain was assessed, and if necessary, analgesia was enhanced with an injection of 50 μg/kg of intraperitoneal buprenorphine.

Twenty-four hours later, rats were split into several groups. CLP animals were randomized into 5 subgroups given a single-blinded i.v. dose of Adrecizumab (2 mg/kg in 1.5 mL) or placebo (1.5 mL of PBS) through the jugular vein and norepinephrine (NE), for either 30 min or continuously as the standard of care for haemodynamic management, or not (CLP, CLP-Adrecizumab, CLP-cNE (with continuous NE), CLP-cNE-Adrecizumab (Adrecizumab + continuous NE), and CLP-NE (with NE infusion for 30 min)). Norepinephrine was administered at a dose of 1 μg/kg/min. The injection of a 1.5-mL bolus of Adrecizumab or placebo mimicked a fluid challenge. Then, the rats were given liquid throughout the experiment with norepinephrine or saline solution.

**Fig. 1:**
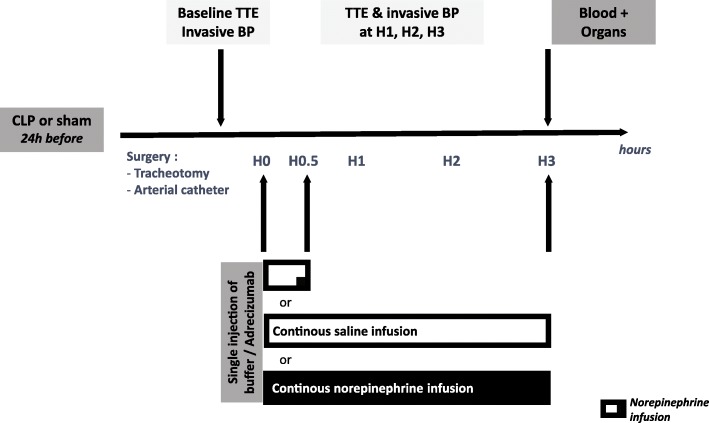
Experimentation protocol

Among CLP rats (*n* = 71), 28% died before administration treatment of either Adrecizumab or placebo and haemodynamic exploration. There were at least seven rats per group who underwent haemodynamic exploration and treatment administration (except for the CLP group with norepinephrine infusion during only 30 min, *n* = 4). Sham animals received neither Adrecizumab nor NE. The experimental protocol is summarized in Fig. 1.

Briefly, 24 h after the CLP procedure, rats were anaesthetized with ketamine hydrochloride (90 mg/kg) and xylazine (9 mg/kg) [21] and placed in the supine position. Animals were intubated with a catheter 16 G and ventilated using a rodent ventilator with respiratory rate = 53.5 × weight^−0.26^ and tidal volume = 6.2 × weight^1.01^. In a 400-g rat, the tidal volume was 2457 μL, and the respiratory rate was 68/min [22].

Rectal temperature was maintained throughout the protocol at 37–37.5 °C by a heating mat. Catheters were inserted into the left jugular vein to administer antibody or placebo and into the right carotid artery to monitor blood pressure.

### Haemodynamics and cardiac function monitoring

Cardiac function was assessed by transthoracic echocardiographic examination at baseline and every hour during the next 3 h of the experiment using a GE Healthcare Vivid 7 Ultrasound System equipped with a high-frequency (14 MHz) linear probe. All examinations were recorded digitally and stored for subsequent offline analysis as described by Milliez et al. [23].

Cardiac dimensions and shortening fraction (SF) were determined in the parasternal long-axis view in M mode of the chest, as described previously [24]. The left ventricular (LV) shortening fraction, taken as an index of LV systolic performance, was calculated as follows:

$$ \mathrm{SF}=\frac{\mathrm{LVED}-\mathrm{LVES}}{\mathrm{LVED}} $$

SF, shortening fraction (%); LVED, left ventricle end-diastolic internal diameter (mm); LVES, left ventricle end systolic diameter (mm).

From the parasternal long-axis B-mode image of the chest allowing measurement of the pulmonary artery diameter, cardiac output was calculated from the ultrasound-derived mean blood flow velocity (mBFV) and diameter measurements of the pulmonary artery according to previously described method [25]. Cardiac output (mL/min) was calculated as follows:

$$ \mathrm{CO}=60\times \left(\mathrm{mBFV}\times \left\{\pi \times {\left(\frac{\mathrm{Dpa}}{2}\right)}^2\right\}\right) $$

CO, cardiac output (mL/min); mBFV, mean blood flow velocity (cm/s), Dpa, pulmonary artery internal diameter (cm).

Invasive blood pressure (BP) measurements were performed after catheter insertion and every hour during the next 3 h. The right carotid artery was catheterized by a polyethylene 50 (PE-50) catheter connected to a pressure head placed at the height of the animal’s heart. Data were recorded by the AcqKnowledge® software (BIOPAC Systems, Inc. USA).

### Assessment of organ inflammatory response and oxidative stress

EDTA blood was collected from the left carotid artery at the end of the experiment and centrifuged at 3500 rpm for 15 min at 4 °C, and plasma was stored at − 80 °C until measurement of several analytes. Bio-ADM was measured as described in [18].

At the end of the protocol, rats were sacrificed, and organs (heart, lung, liver, and left kidney) were weighed. The heart was transversely divided into two parts. The base was embedded into Tissue-Tek optimal cutting temperature (OCT) compound (Sakura Finetek, France) and frozen in liquid nitrogen and stored at − 80 °C until use for dihydroethidium (DHE) staining; the other part of the heart was snap-frozen in liquid nitrogen for RT-PCR and Western blot analysis.

Other organ specimens (lung, liver, kidney, brain, aorta, and muscle) were collected and snap-frozen in liquid nitrogen. All samples were stored at − 80 °C until further analysis.

#### Gene expression analysis

Total RNA was isolated from tissues using the RNeasy Mini Kit® (Qiagen, Courtaboeuf, France) according to the manufacturer’s instructions and reverse transcribed using QuantiTect® Reverse Transcription (Qiagen, Courtaboeuf, France). Then, real-time polymerase chain reaction was performed with a LightCycler 96 system (Roche Diagnostics, Meylan, France) using the FastStart Essential DNA Green Master® (Roche Diagnostics, Meylan, France). mRNA levels for genes of interest were normalized to that of glyceraldehyde-3-phosphate dehydrogenase (GAPDH), expressed as the relative change compared with the control samples. The sequences of the primers used are reported in Additional file 1: Table S1.

#### Protein analysis

For Western blot analysis, tissues were homogenized in cell lysis buffer (50 mM Tris HCl at pH 7.4, 1 mM EDTA, and 150 mM NaCl). After centrifugation, soluble proteins were quantified using the Pierce BCA Protein Assay Kit (Thermo Fisher Scientific, Courtaboeuf, France). Proteins (30 μg) were separated on 10–12% SDS-PAGE gels and transferred onto nitrocellulose membranes (Protan, Paris, France). Blots were probed overnight at 4 °C with the following primary antibodies directed against the following: phosphorylated and total Akt (Ser473) (1:1000; #9271 and #9272; Cell Signaling, Ozyme, France), p62 (1:1000; ab56416; Abcam, UK), HIF1α (1:1000; PAI 16601; Thermo Scientific, MA, USA), and GAPDH (1:5000; Millipore, Molsheim, France). Blots were incubated with goat anti-rabbit (1:5000; Sigma-Aldrich) or sheep anti-mouse peroxidase-conjugated antibodies (1:10,000; GE Healthcare) for 1 h at room temperature. Chemiluminescent signals (ECL Plus; GE Healthcare) were recorded using an LAS 3000 system (Fuji, Courbevoie, France) and were quantified using MultiGauge V2.02 software (Fuji).

The results are expressed as arbitrary units (AU) obtained from the ratio between the densitometric units of the protein under study and the GAPDH densitometric value.

#### Histological and histochemical analyses

Seven micrometer cross-sections were stained with haematoxylin and eosin and examined by bright-field microscopy at × 20 magnification.

Cardiac cryostat cross-sections (7 μm) of the ventricles were incubated with dihydroethidium (DHE; Sigma-Aldrich) (37 μM) for 30 min in a dark humidified chamber [26]. Acquisition of fluorescent images of ethidium bromide with a Leica fluorescence microscope was performed under identical settings regardless of the block tissue. The stained area was measured with IPLab software and is expressed as a percentage of area of interest (% of ROI).

### Statistical analysis

Data are presented as the mean ± SEM. Statistical analysis was performed using GraphPad Prism version 5.0 (GraphPad Software Inc., San Diego, CA, USA). Comparison between groups was performed by two-way ANOVA or a Kruskal-Wallis test followed by Dunn’s multiple comparison test as appropriate. The Mann-Whitney test was used to compare baseline haemodynamic parameters. A *p* value < 0.05 was considered statistically significant.

## Results

At the time of haemodynamic resuscitation, clinical signs of sepsis (reduced motor activity, lethargy, shivering, piloerection, and hunched posture) were only present in CLP rats as expected. Furthermore, post-mortem examination of the abdominal cavity of all CLP rats showed varying degrees of peritonitis with a grey-black dilated caecum and purulent and malodorous peritoneal fluid. There was no difference in the heart, lung, liver, and left kidney weights between sham and CLP rats (Additional file 2: Table S2).

### Benefits of Adrecizumab on haemodynamics

**Fig. 2:**
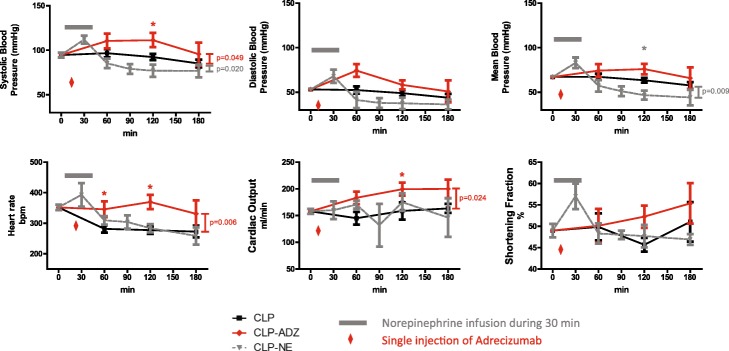
Haemodynamic parameters after sepsis induction, until 3 h after the injection of Adrecizumab or placebo. Haemodynamic parameters measured 24 h after sepsis induction by CLP and then 60, 120, and 180 min after the injection of Adrecizumab or placebo. CLP rats are represented by the black line, CLP-Adrecizumab rats by the red line, and CLP-NE rats by the grey line. Two-way ANOVA was used. *p < 0.05

Before initiation of haemodynamic resuscitation, all CLP rats presented with altered haemodynamics, including a markedly lower mean BP (68 ± 2 vs 88 ± 3 mmHg, *p* < 0.0001) and a marked increase in cardiac output (0.160 ± 0.005 vs 0.133 ± 0.008 mL/min, *p* = 0.018), compared to sham rats (Additional file 3: Figure S1). In untreated CLP rats, haemodynamics remained altered during the 3 h of experimentation (Fig. 2).

A single dose of Adrecizumab (CLP-Adrecizumab rats) without norepinephrine rapidly increased systolic blood pressure (*p* = 0.049 vs untreated CLP). Adrecizumab injection also tended to improve diastolic and mean blood pressures and LV shortening fraction, although these did not reach statistical significance due to a possible type 2 error (Fig. 2).

In addition, cardiac output significantly increased during the 3 h after single-dose administration of Adrecizumab compared to that of untreated CLP rats (*p* = 0.006, Fig. 2). Sham rats were bradycardic compared to CLP rats (*p* = 0.024, Fig. 2).

During the protocol, restoration of systolic blood pressure and improvement in cardiac output were similar in CLP rats receiving continuous NE infusion and those receiving a single dose of Adrecizumab (Additional file 4: Figure S2). Furthermore, the addition of a single injection of Adrecizumab to continuous NE infusion had a similar effect as NE on haemodynamic parameters and did not lead to further unwanted vasoconstriction. Additional file 4: Figure S2 shows haemodynamic parameters measured 24 h after sepsis induction by CLP and 120 min after a single injection of Adrecizumab or placebo.

Of note, short-term (30 min) administration of NE only transiently improved haemodynamics, and parameters either returned to baseline values (e.g., cardiac output and heart rate) or worsened (e.g., blood pressure) compared to baseline after stopping NE infusion (Fig. 2).

### Adrecizumab and metabolic changes in sepsis

**Fig. 3:**
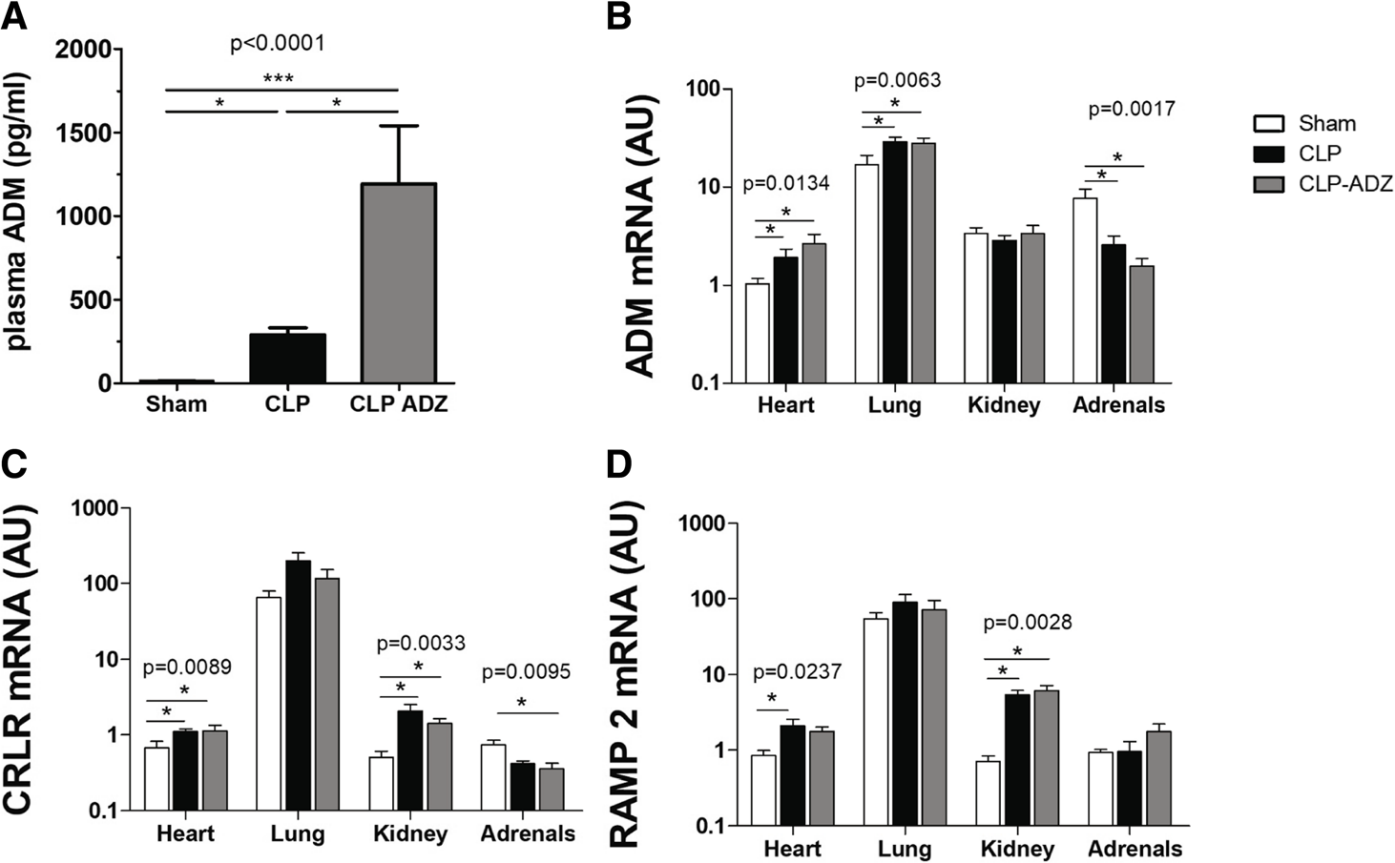
Effect of Adrecizumab on the adrenomedullin pathway. **a** Adrenomedullin plasma level (pg / mL). **b**–**d** Expression of adrenomedullin (ADM) mRNA and its receptors (CRLR, RAMP 1 and 2) in the heart, lungs, kidneys, and adrenals. These measurements were performed 3 h after the Adrecizumab injection and 24 h after the induction of sepsis. Kruskal-Wallis test followed by Dunn’s multiple comparison test was used. *p < 0.05 and ***p < 0.001

Concerning circulating bio-ADM, levels were low in sham animals (14.6 ± 2.1 pg/mL), while a strong elevation was observed in untreated CLP rats (289.7 ± 42.2 pg/mL, *p* < 0.05 vs sham; Fig. 3). Administration of Adrecizumab further increased plasma bio-ADM concentration (1193 ± 349.8 pg/mL, *p* < 0.05 vs sham; Fig. 3).

Myocardial and lung ADM levels were increased in the untreated CLP vs sham groups (heart 2.17 ± 0.4 vs 0.9 ± 0.1, *p* < 0.05; lung 29.1 ± 3.1 vs 13.4 ± 2.2, *p* < 0.05; Fig. 3) and remained high 3 h after single Adrecizumab injection (heart 2.64 ± 0.6, *p* < 0.05 vs sham; lung 28.1 ± 3.6, *p* < 0.05 vs sham). In contrast, adrenal expression of ADM decreased in both untreated CLP and CLP-Adrecizumab rats versus sham rats (respectively 2.57 ± 0.6, 1.58 ± 0.3 vs 7.67 ± 1.9, *p* < 0.05). No change in ADM expression was observed in the lung.

Expression of the ADM receptor components CRLR and RAMP2 was by far highest in the lung, 50–100-fold higher than in the heart, kidneys, and adrenals. Myocardial and kidney expression of CRLR was increased in the untreated CLP vs sham groups (heart 1.11 ± 0.1 vs 0.54 ± 0.1, *p* < 0.05; kidney 2.08 ± 0.5 vs 0.51 ± 0.1, *p* < 0.05; Fig. 3) and remained high 3 h after single Adrecizumab injection (heart 1.14 ± 0.2, *p* < 0.05 vs sham; kidney 1.42 ± 0.2, *p* < 0.05 vs sham). In contrast, adrenal expression of CRLR decreased significantly in the CLP-Adrecizumab vs sham groups (0.36 ± 0.06 vs 0.74 ± 0.11, *p* < 0.05) and non-significantly in the untreated CLP group (0.42 ± 04). No change in CRLR expression was observed in the lung.

Myocardial and kidney expression of RAMP 2 was increased in the untreated CLP group vs the sham group (heart 2.12 ± 0.5 vs 0.89 ± 0.2, *p* < 0.05; kidney 5.42 ± 0.8 vs 0.71 ± 0.1, *p* < 0.05; Fig. 3) and remained high 3 h after single Adrecizumab injection (heart 1.77 ± 0.3, ns vs sham; kidney 6.14 ± 1.1, *p* < 0.05 vs sham). No change in RAMP 2 expression was observed in the lung and adrenals.

**Fig. 4:**
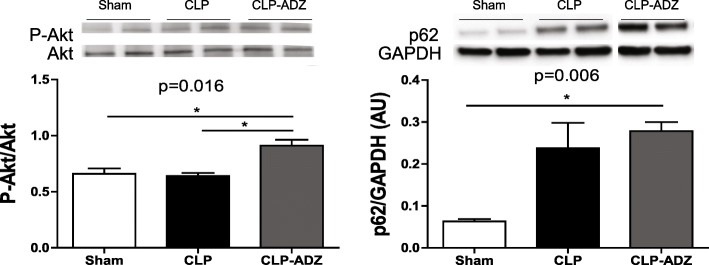
Activation of the survival pathway in septic rats treated with Adrecizumab. Western blot analyses of cardiac P-Akt/Akt and p62. Kruskal-Wallis test followed by Dunn’s multiple comparison test was used. *p < 0.05

Regarding myocardial inflammatory markers, the expression of the proinflammatory marker TNFα, the anti-inflammatory marker IL-10 and CD68, a marker of macrophage activation, were all increased in CLP rats. Myocardial levels of these markers were not altered in CLP-Adrecizumab rats. Moreover, myocardial BNP mRNA was upregulated in untreated CLP rats and remained high in CLP-Adrecizumab. In addition, p62, an inflammatory marker, increased in the hearts of untreated CLP and CLP-Adrecizumab rats compared with that in the sham rats (Fig. 4). Additional file 5: Figure S3 also shows that myocardial BNP mRNA was upregulated in the CLP group and remained high in the CLP-Adrecizumab group.

Figure 4 shows that the Akt phosphorylation level, a myocardial survival pathway, was markedly increased in the myocardium of CLP-Adrecizumab rats 24 h after the onset of sepsis (*p* < 0.05).

**Fig. 5:**
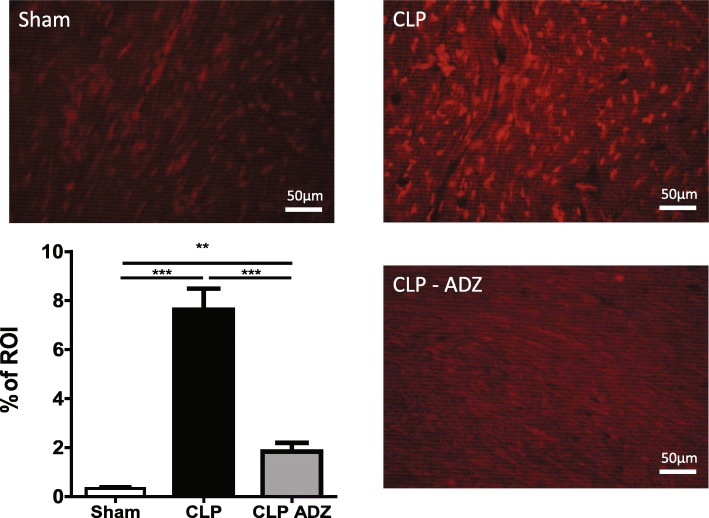
Adrecizumab decreases ROS production. Adrecizumab decreased ROS production in the septic myocardium within 3 h. Dihydroethidium (DHE; Sigma-Aldrich) staining was used to evaluate the in situ levels of superoxide anion in the myocardium. Data are expressed as a percentage of region of interest (percent of ROI). Kruskal-Wallis test followed by Dunn’s multiple comparison test was used. *p < 0.05 and ***p < 0.001

Concerning myocardial oxidative stress, the CLP-induced a 10-fold elevation of DHE that was blunted in CLP-Adrecizumab rats (*p* < 0.05) (Fig. 5).

## Discussion

The present study showed that a single therapeutic injection of Adrecizumab in septic rats induced rapid haemodynamic benefits and a marked reduction in myocardial oxidative stress. Indeed, antibodies directed against ADM, an endogenous vasodilator peptide, had similar haemodynamics to continuous administration of norepinephrine.

Our study demonstrated that in a model of sepsis, a single injection of Adrecizumab rapidly restored blood pressure and cardiac output. ADM has been proposed to be one of the pivotal mediators of vascular dysfunction in sepsis. In patients resuscitated for sepsis or septic shock, the plasma bio-ADM level was always related to prognosis [13]. The highest concentration of bio-ADM was associated with the need for vasoconstrictors. As recently described, circulating bio-ADM easily diffuses from the lumen of the vessels to the interstitium to act on vascular smooth muscle cells and reduce vascular tone [11]. Adrecizumab was described to improve blood pressure when given as preventive therapy before induction of sepsis [20]. Herein, we showed that a single injection of Adrecizumab restored blood pressure 24 h after induction of peritonitis and septic shock in rats. The early benefit of a single injection of Adrecizumab on blood pressure (Fig. 2) was likely related to the rapid binding of Adrecizumab to plasma bio-ADM, hence preventing its diffusion to the interstitium.

Our study further showed that a single therapeutic injection of Adrecizumab was associated with a sustained improvement in cardiac output in septic rats. This is the first demonstration that Adrecizumab, likely acting as a scavenger of circulating bio-ADM, not only improves blood pressure but also improves systemic perfusion. Improvement in cardiac output might be related, at least partially, to the higher heart rate after single administration of Adrecizumab. The data also indicated that Adrecizumab improved the left ventricular shortening fraction, though not significantly, in septic rats.

In septic shock patients, haemodynamics, especially blood pressure, is usually restored by continuous administration of vasopressors such as catecholamine, vasopressin, or angiotensin [27]. In the present study, we restored blood pressure and improved cardiac output with a single administration of Adrecizumab at levels similar to continuous administration of norepinephrine. This novel approach might be safer as it avoids the long-lasting administration of vasopressors, which is possibly associated with deleterious effects on outcome [3]. Therefore, norepinephrine substitution by Adrecizumab might be of interest. Our data showed that the Adrecizumab benefits on blood pressure at a dose of 2 mg/kg might be smaller after 2 h, although the benefits on cardiac output appeared to be maintained. Further studies should be conducted at higher doses and with increased animal monitoring to avoid the bias of potential hyporesponsiveness to catecholamines and to assess the long-term effect of Adrecizumab. Hence, the present preclinical work confirms and extends the short-term safety and efficacy profile of the non-neutralizing ADM-binding antibody Adrecizumab in line with the improved renal function and survival previously described [18, 19]. These results led to the pursuit of this programme in human septic patients. Adrecizumab is still under investigation. A phase 2 trial, AdrenOSS-2, started in December 2017 to assess the safety and efficacy of a single injection of Adrecizumab (2 or 4 mg/kg) in patients with septic shock (NCT 03085758). The AdrenOSS-2 trial is one of the first personalized medicine trials in septic shock patients. Patient selection is guided not only by clinical parameters but also by biomarker-guided measurements of circulating biologically active ADM concentrations at admission. Adrecizumab will be given only to patients who need it.

The source of ADM in plasma includes production by many cells, including endothelial cells, vascular smooth muscle cells, monocytes, renal parenchymal cells, and macrophages. Studies on rat endothelial cells have shown that ADM is not stored but rather constitutively produced and that endothelial cells secrete ADM at a higher rate than vascular smooth muscle cells [18]. Our data confirmed that rats treated with Adrecizumab have an increase in plasma ADM [18].

Regarding CRLR, RAMP-2, and ADM expression in various tissues, our data confirmed that the ADM pathway is highly present in the lungs compared to other organs, including the heart, kidney, and adrenal glands [28, 29]. Twenty-four hours of peritonitis and septic shock induced changes in the ADM pathway, with greater expression of ADM in the heart and lung and of CRLR and RAMP 2 in the heart and kidney. In adrenals, where ADM was first described [4], sepsis decreased ADM and CRLR expression. The single injection of Adrecizumab had no effect on the ADM pathway. Most studies refer to “ADM receptors” without specifying which receptor is specifically activated. Therefore, we focused only on the ADM receptor composed of CRLR and RAMP 2.

Peritonitis and septic shock upregulated inflammation and survival pathways in the heart and in other organs. These changes were unaffected as early as 3 h after a single Adrecizumab injection. The observation time was likely too short to see any significant changes in the ADM pathway and tissue inflammation, and a longer observation period is needed. In contrast, single Adrecizumab injection succeeded in rapidly and markedly reducing myocardial oxidative stress. The mechanisms of Adrecizumab’s benefits on tissue oxidative stress are not fully understood, although the antiapoptotic and antioxidative properties previously described might be involved [11, 18]. The improvement in myocardial function may also be related to the Adrecizumab-induced marked reduction in myocardial oxidative stress. The latter is known to improve both systolic and diastolic function that might contribute to the sustained benefit in cardiac output following a single administration of Adrecizumab in septic rats [30, 31].

## Conclusion

Therapeutic treatment with the ADM-binding antibody Adrecizumab improves short-term haemodynamic parameters and attenuates myocardial oxidative stress in rat polymicrobial sepsis. Currently, a proof-of-concept and dose-finding phase II trial is ongoing in patients with septic shock and elevated concentrations of circulating bio-ADM.

## Additional files

Additional file 1: **Table S1.** The sequences of primers used for mRNA analysis. (DOC 32 kb)

Additional file 2: **Table S2.** Organs weight/body weight. (DOC 35 kb)

Additional file 3: **Figure S1.** Haemodynamics of the CLP model Haemodynamics 24 h after CLP and prior to antibody injection. A Average arterial pressure (mmHg). B Heart rate (bpm). C Fraction of shortening. D Cardiac flow (ml/min). The Mann-Whitney test was used. (EPS 2083 kb)

Additional file 4: **Figure S2.** Comparison of the haemodynamic effect of Adrecizumab and norepinephrine. Haemodynamic parameters measured 24 h after sepsis induction by CLP and 120 min after a single injection of Adrecizumab or placebo. cNE indicates continuous administration of norepinephrine. Kruskal-Wallis test followed by Dunn’s multiple comparison test was used. (EPS 2038 kb)

Additional file 5: **Figure S3.** Cardiac mRNA expression of inflammation and BNP A and B. Expression of cardiac cytokine mRNAs. C and D. Expression of BNP and CD68 cardiac mRNAs. These measurements were performed 3 h after the Adrecizumab injection and 24 h after the induction of sepsis. Kruskal-Wallis test followed by Dunn’s multiple comparison test was used. *p < 0.05. (EPS 4399 kb)

## Abbreviations

ADM: Adrenomedullin
bio-ADM: Biologically active adrenomedullin
CLP: Caecal ligation and puncture
cNE: Continuous norepinephrine
CO: Cardiac output
CRLR: Calcitonin-receptor-like receptor
DHE: Dihydroethidium
Dpa: Pulmonary artery internal diameter
GAPDH: Glyceraldehyde-3-phosphate dehydrogenase
LV: Left ventricular
LVED: Left ventricular end-diastolic internal diameter
LVES: Left ventricular end-systolic internal diameter
mBFV: Mean blood flow velocity
NE: Norepinephrine
RAMP: Receptor activity modifying protein
SF: Shortening fraction
