# Supplementary material for: Adrecizumab, a non-neutralizing anti-adrenomedullin antibody, improves haemodynamics and attenuates myocardial oxidative stress in septic rats
Source: Intensive Care Med Exp. 2019 May 15;7:25. doi: 10.1186/s40635-019-0255-0 (PMC6520420; doi:10.1186/s40635-019-0255-0)
Supplement: Supplementary file 1 — Table S1. The sequences of primers used for mRNA analysis. (DOC 32 kb) [file 40635_2019_255_MOESM1_ESM.doc]

**Table S1.** The sequences of primers used for mRNA analysis

|  | **Gene** | **Access GenBank** | **Forward sequence** | **Reverse sequence** |
| --- | --- | --- | --- | --- |
| **Adrenomedullin** | *Adm* | NM_012715.1 | TTCTCATCGCAGTCAGTCTTGG | CGCTTGTAGTTCCCTCTTCCC |
| **BNP** | *Nppb* | NM_031545.1 | TAGCCAGTCTCCAGAACAATCCA | AAACAACCTCAGCCCGTCAC |
| **CD68** | *Cd68* | NM_001031638.1 | GCCCTCACCAAGTCCTAGTC | GATGTCGGTCCTGTTTGAATCCA |
| **CRLR** | *Calcrl* | NM_012717.1 | TCAGCTCAGACACTCATCTCCTC | CACCTCCTCAGCAACCTTTCC |
| **GAPDH** | *Gapdh* | NM_017008.4 | GTTCAACGGCACAGTCAAGG | ACTCCACGACATACTCAGCAC |
| **IL10** | *IL10* | NM_012854.2 | CCTGCTCTTACTGGCTGGAG | TGTCCAGCTGGTCCTTCTTT |
| **RAMP2** | *Ramp2* | NM_031646.1 | TTGTGGTGTGGAGGAGTAAAGAC | GTGGGAAGGATGGGAGTAAGTG |
| **TNF** | *Tnf* | NM_012675.3 | ACTCCCAGAAAAGCAAGCAA | CGAGCAGGAATGAGAAGAGG |
