# Supplementary material for: Adrecizumab, a non-neutralizing anti-adrenomedullin antibody, improves haemodynamics and attenuates myocardial oxidative stress in septic rats
Source: Intensive Care Med Exp. 2019 May 15;7:25. doi: 10.1186/s40635-019-0255-0 (PMC6520420; doi:10.1186/s40635-019-0255-0)
Supplement: Supplementary file 2 — Table S2. Organs weight/body weight. (DOC 35 kb) [file 40635_2019_255_MOESM2_ESM.doc]

**Table S2.** Organs weight/body weight

|  | **Sham** | **CLP** | **CLP**  **-ADZ** | **CLP**  **-cNE** | **CLP**  **-cNE**  **-ADZ** | **CLP**  **-NE** | ***p value***  ***(Kruskal-Wallis)*** |
| --- | --- | --- | --- | --- | --- | --- | --- |
| **Heart/body weight * 1000** | 3.33 ± 0.09 | 3.17 ± 0.08 | 3.13 ± 0.05 | 3.28 ± 0.06 | 3.26 ± 0.06 | 3.07 ± 0.05 | ns |
| **Lung/body weight * 1000** | 4.02 ± 0.15 | 3.92 ± 0.08 | 4.13 ± 0.11 | 3.86 ± 0.11 | 3.79 ± 0.20 | 4.83 ± 0.79 | ns |
| **Kidney/body weight * 1000** | 3.51 ± 0.14 | 3.97 ± 0.13 | 3.87 ± 0.18 | 3.74 ± 0.18 | 3.80 ± 0.19 | 3.51 ± 0.25 | ns |
| **Liver/body weight * 1000** | 37.53 ± 1.33 | 36.80 ± 1.30 | 35.10 ± 0.95 | 35.16 ± 1.36 | 32.93 ± 1.01 | 33.16 ± 0.64 | ns |

Data are presented as mean ± Standard Error of Mean (SEM). Comparison between groups was performed by Kruskal Wallis analysis followed by Dunns test. A *p*-value of < 0.05 was considered statistically significant.

**CLP**: cecal ligation and puncture**, CLP-ADZ**: CLP-Adrecizumab, **CLP-cNE**: CLP-continuous Norepinephrine infusion, **CLP-cNE-ADZ**: CLP-continuous Norepinephrine infusion-Adrecizumab, **CLP-NE**: CLP- Norepinephrine infusion during 30 min.
